# Metabolomic profiling reveals root exudation pattern as regulated by phosphorus and light in contrasting rice cultivars

**DOI:** 10.1186/s12870-026-08527-5

**Published:** 2026-03-26

**Authors:** Lin Guo, Fengyue Yu, Yixuan Zhu, Xueyun Deng, Yongjun Zeng, Xueming Tan

**Affiliations:** 1 Ministry of Education Key Laboratory of Crop Physiology, Ecology and Genetic Breeding, College of Agronomy, Jiangxi Agricultural University, Nanchang, 330045 China; 2 College of Chemistry and Materials, Jiangxi Agricultural University, Nanchang, 330045 China

**Keywords:** Rice, Cultivar, Phosphorus supply, Light intensity, Root exudation

## Abstract

Phosphorus (P) fixation in soils constrains its bioavailability, driving the evolution of adaptive strategies in plants, such as root morphology plasticity and exudation. This study systematically compared changes in root exudation pattern in a low-P-tolerant rice cultivar “Dalixiang” and a P-susceptible cultivar “Meixiangzhan” under different light intensities and P supplies. The results showed that, (1) Under P deficiency, Dalixiang exhibited a significantly increased root-to-shoot ratio, whereas Meixiangzhan showed a weaker ability to cope with P deficiency and low light. (2) Light intensity exerts a quantitatively greater influence on root exudation profiles than P supply, as evidenced by both the number of differential metabolites (301 vs. 185) and the magnitude of fold changes in key compounds. (3) The tolerant cultivar exhibited greater metabolic flexibility, releasing a broader spectrum of exudates across environments. Key pathways, including amino acid metabolism, were reconfigured by both stimuli. (4) Several previously unreported metabolites co-regulated by light intensity and P availability demonstrated their functional relevance. Notably, α-methylene-γ-butyrolactone significantly promoted root and shoot growth under P deficiency, while citraconic acid and xanthoxic acid enhanced P uptake. Our findings establish that light and P status dictates root physiological and metabolic traits, ultimately modulating rhizosphere P mobilization. This work provides a mechanistic framework for improving P-use efficiency in crops by leveraging the interplay between light management and root-mediated processes.

**Supplementary Information:**

The online version contains supplementary material available at 10.1186/s12870-026-08527-5.

## Key Message

Light overrides P availability in reshaping root exudation patterns in rice. A P-tolerant cultivar exhibits greater metabolic flexibility, and several key exudates enhance plant growth under P deficiency.

**Supplementary Information:**

The online version contains supplementary material available at 10.1186/s12870-026-08527-5.

## Introduction

Phosphorus (P) is a vital and structurally irreplaceable element in biomolecules including nucleic acids, ATP, and phospholipids [1]. In the vast red soil regions of southern China, however, much of the soil P is highly fixed by iron and aluminum oxides, which drastically reduces its bioavailability [2]. This process severely limits the P acquisition efficiency of staple crops such as rice (*Oryza sativa* L.), thereby impairing their yield potential.

To address this challenge, plants have evolved sophisticated strategies to mobilize rhizosphere P through root exudates [3]. Based on molecular weight, root exudates are broadly classified into two categories: (i) high-molecular-weight compounds, primarily comprising mucilage and diverse extracellular enzymes; and (ii) low-molecular-weight compounds, encompassing organic acids, amino acids, sugars, phenolic compounds, phytosiderophores (PS), and other secondary metabolites [4]. Among these, low-molecular-weight exudates have attracted considerable attention due to their multifunctional ecological roles: they not only facilitate the assembly of rhizosphere microbial communities and alleviate aluminum toxicity via chelation [5], but also mobilize poorly soluble nutrients (P, Fe, Zn, etc.) and accelerate mineral weathering processes [6]. Taking citrate as an example, it employs a proton cotransport mechanism to acidify the rhizosphere, effectively promoting the release of P from Fe/Al-P complexes [7].

Root exudation patterns exhibit remarkable plasticity [8], with their quantitative and qualitative profiles being regulated by multiple factors, including plant species/cultivar, developmental stage, environmental cues (e.g., light intensity and temperature), and nutritional status [9]. Distinct exudation signatures are observed among plant species and even between cultivars of the same species under nutrient deficiency [10]. Rice, as a staple food crop, demonstrates significant cultivar variation in the composition and dynamics of root exudates [11]. Deciphering the alterations in root morphology and exudate profiles of low-P-tolerant rice cultivars under varying P availability is crucial for elucidating the key mechanisms underlying their adaptation to P deficiency, thereby providing a theoretical foundation for P-efficient rice cultivation.

Notably, plants allocate 20–60% of photosynthetically fixed carbon to belowground compartments [12], with approximately 5–30% ultimately released into the rhizosphere as root exudates [13]. As a key environmental regulator of photosynthetic carbon assimilation, light intensity profoundly shapes exudation dynamics by influencing photo-assimilate transport and respiratory metabolism. Diurnal rhythm studies reveal significantly higher rhizosphere carbon efflux during daylight hours compared to nocturnal periods [14], a phenomenon intrinsically linked to light-mediated regulation of carbon metabolism. Mechanistic investigations demonstrated that light intensity remodels root physiology through sucrose signaling pathways [15]. Critically, light intensity may interact with nutrient stress—particularly P deficiency—to shape root exudation strategies. Under P limitation, plants often enhance exudation of organic acids and phosphatases to mobilize insoluble P (Gerke 2024). However, this process is carbon-costly and thus likely modulated by photo-assimilate supply. For instance, under P deficiency, white lupin (*Lupinus albus* L.) allocates 40% of net fixed carbon to citrate and malate exudation [16], leading us to hypothesize that enhanced light intensity not only modulates total exudation flux via altered photo-assimilate partitioning but may also significantly augment P acquisition efficiency. Importantly, even under P-sufficient conditions, sucrose accumulation in roots can activate transcriptional networks governing organic acid biosynthesis [17]. These findings collectively suggest that in environments with deficient P, light intensity exerted a profound and consistent influence on root exudation patterns.

Therefore, understanding how light intensity modulates root exudation under varying P supply is essential to elucidate the carbon-nutrient trade-offs that underlie plant adaptation to low-P environments. Building upon this conceptual framework, we propose a novel hypothesis: under P-deficient conditions, elevated light intensity enhances photosynthetic carbon assimilation, thereby reprogramming root development and exudation patterns. Through establishing a precisely controlled experimental system coupling light intensity with P supply, this study aims to: (1) determine whether light or P availability is the dominant driver of root exudation reprogramming; (2) identify novel, functionally relevant metabolites within the exudate profile that respond to light and P interplay; and (3) quantify their efficacy in alleviating P stress. These findings will establish a theoretical foundation for developing precision field management strategies based on light-nutrient co-regulation.

## Materials and methods

### Experimental design

We used two rice cultivars for the experiment: the low-P-tolerant Dalixiang (D) and the P-susceptible Meixiangzhan (M). Dalixiang exhibited higher biomass, P uptake, and root acid phosphatase activity under P deficiency, while Meixiangzhan showed stunted growth and reduced P content [18]. After surface-sterilization in 3% (v/v) sodium hypochlorite for 20 min, the seeds were rinsed three times with distilled water. The healthy, plump seeds were germinated in bags soaked in distilled water at 30 °C until radicles appeared. They were then transferred to moist towels and returned to 30 °C until coleoptiles attained a length of 5 mm. The germinated seeds were grown in 96-cell black hydroponic boxes (20 mm aperture) in an artificial climate chamber set to a 10/14 h light/dark cycle, a PPFD of 800 µmol m⁻² s⁻¹, temperatures of 25/22°C (day/night), 60 ± 5% relative humidity, and 400 ± 20 µmol mol⁻¹ CO₂. Oxygenation was maintained via gentle aeration (air pumps) and the use of opaque boxes to minimize algal growth.

Treatment involving light intensity and P levels was initiated at the two-leaf stage, which represents the onset of active root development and carbon allocation in rice. Following the onset of treatments, healthy seedlings of uniform size were transplanted into 6-cell black hydroponic boxes (20 mm aperture) at a density of 12 plants per pot (2 plants per cell). The root systems were kept in the dark as the openings in the boxes were plugged by seedling-holding sponges. The boxes contained 2 L of a 1/4-strength nutrient solution deficient in P. The nutrient solution was prepared with the following composition: Ca(NO₃)₂·4 H₂O (2500 µM), KCl (1000 µM), K₂SO₄ (1000 µM), MgSO₄·7 H₂O (750 µM), H₃BO₃ (30 µM), MnSO₄·H₂O (2.5 µM), ZnSO₄·7 H₂O (1 µM), CuSO₄·5 H₂O (1 µM), (NH₄)₆Mo₇O₂₄·4 H₂O (0.3 µM), and Fe-EDDHA (50 µM). Two distinct P regimes were defined: a sufficient-P (SP) treatment with 500 µM NaH₂PO₄·2 H₂O, and a deficient-P (DP) treatment. To avoid a rapid depletion of P content in conventional low-P nutrient solutions—where concentrations often drop below detectable limits within a very short period (e.g., a few hours), leaving plants in a state of P starvation for most of the cultivation cycle, the DP condition in this study was characterized by the use of 1 g L^− 1^ granular phosphate rock (1–5 mm particle size) to mimic the continuous and stable low-P supply environment found in actual soil. The granular material was contained within 30 μm pore-size tea bags to eliminate direct adhesion to roots. P concentration was monitored daily throughout each 7‑day cycle. In a weakly acidic hydroponic environment (pH 5.6–6.5), a stable and sustained low‑P concentration (0.1–0.4 µg L⁻¹) was maintained, effectively simulating the continuity and stability of low‑P stress in soil [18]. When the seedlings reached the three-leaf stage, they were nourished with a half-strength nutrient solution for one week before being switched to a full-strength solution [19]. The nutrient solution was renewed weekly, and the pH was adjusted daily to 5.5 ± 0.2 using dilute H₂SO₄.

The experiment featured two light regimes: high light (HL at 800 µmol m⁻² s⁻¹ PPFD) and low light (LL at 300 µmol m⁻² s⁻¹ PPFD). The HL PPFD was delivered by the growth chamber lights over a 10-hour photoperiod (08:00–18:00), whereas the LL condition was created using black shade nets with 37.5% ± 2.5% transmittance. A completely randomized design with three biological replicates per treatment was employed. Weekly rotation of hydroponic boxes minimized micro-environmental variations, and PPFD was consistently monitored and calibrated with an LI-250 A light meter (LI-COR, U.S.A.). Other growth conditions were uniformly maintained. The efficacy of seven selected differential metabolites in alleviating P deficiency was evaluated using the P-susceptible cultivar Meixiangzhan under high light conditions (800 µmol m⁻² s⁻¹ PPFD). The experiment followed the same hydroponic system and P-deficient (DP) treatment as described above. The compounds were dissolved appropriately and added to the nutrient solution at the final concentrations 1 µM. The nutrient solution containing the metabolites was replaced every three days to maintain stable activity. After 14 days of treatment, P content was measured as described in 2.3.1.

### Sample collection

#### Collection of root exudates

Root exudates were collected from five-leaf stage plants using an immersion method [20]. Seedlings were carefully transferred to a 500 µM Ca(NO₃)₂ solution and immersed for 12 h to stabilize the root ion environment and minimize non-specific exudation. Following immersion, root systems were thoroughly rinsed three times with deionized water to remove surface-adhered ions and residues. The cleaned roots were then immersed in 200 mL of deionized water (collection solution) for a 2-hour exudate collection. This collection was conducted under the specific light regime (HL or LL) corresponding to the treatment of the plants, while the roots themselves remained in the dark within the opaque hydroponic boxes. Collection commenced at 10:00 AM (after 2 h of illumination) to standardize timing and mitigate diurnal variation effects on exudate release. The exudate solution was immediately filtered through filter paper (MN615, 1/4 Ø90mm, MACHEREY-NAGEL GmbH, Düren, Germany) and rapidly frozen in liquid nitrogen. Lyophilized samples were reconstituted in a methanol-water mixture (50:50, v/v) for 1 h, followed by concentration to complete dryness using a rotary vacuum concentrator (Eppendorf 5305, Shanghai, China). The dry weight was recorded, and all samples were stored at -20 °C pending subsequent HPLC-MS analysis.

#### Sampling of rice root systems

We harvested the plants after root exudate collection and separated the roots from shoots with sterile scissors. The roots were then thoroughly rinsed with deionized water, blotted dry on filter paper, and immediately flash-frozen in liquid nitrogen. Root samples were flash-frozen in liquid nitrogen and finally kept at − 20 °C for future root morphology analysis [21].

### Sample analysis

#### Determination P content

2.3.1 Dried samples (80 °C, constant mass) were pulverized in a cryogenic grinder. Approximately 0.3 g of the homogenized powder was then digested for P analysis using 4 mL of 65% HNO₃ and 2 mL of 30% H₂O₂ in a programmable digestion system. The temperature protocol involved a 2-hour ramp to 120 °C and a 4-hour hold at 280 °C. The digested samples were analyzed for total P content via the molybdate-vanadate method, measuring absorbance at 440 nm on a Shimadzu UV-3600 spectrophotometer [22].

#### Root morphology analysis

Prior to morphological analysis, root samples from each treatment were thawed at 4 °C for about 2 h [21]. The roots were then carefully spread in deionized water within a transparent scanning tray to minimize overlap. Subsequent image acquisition was performed with an EPSON V700 flatbed scanner, and the obtained images were processed by WinRHIZO Pro software to determine morphological traits such as length, diameter, volume, tip count, and surface area. All scans and analyses were conducted in triplicate under standardized ambient conditions of 25 ± 1 °C and 60 ± 5% relative humidity.

#### HPLC-MS-based metabolomic profiling of root exudates

Lyophilized exudates were reconstituted in 1 mL of methanol–water (50:50, v/v) solution followed by vortex mixing for 1 h to ensure complete dissolution. The samples were initially centrifuged at 4,800 rpm (approximately 2,500 × g) for 10 min to remove insoluble particulates. The resulting supernatant was subjected to a second centrifugation step at 14,000 rpm (approximately 16,000 × g) for 10 min to further clarify the solution. A 10 µL aliquot of the final supernatant was injected for HPLC–MS analysis. Metabolite separation was performed using a high-performance liquid chromatography system (Prostar 210, Varian, Darmstadt, Germany) equipped with a reversed-phase Polaris C18-Ether column (100 × 2 mm, 3 μm particle size; Varian) protected by a C18 guard column and maintained at 40 °C. The mobile phase consisted of (A) distilled water: acetonitrile (95:5, v/v) and (B) methanol, both supplemented with 7 mM acetic acid. Detection was carried out using an ion trap mass spectrometer (500 MS, Varian) coupled with an electrospray ionization (ESI) source. Data were acquired in alternating positive and negative ionization modes. In positive ion mode, full scans were acquired across an m/z range of 50–1,000. In negative ion mode, segmented scanning was applied covering m/z 50–400 and 400–1,000. The scan rate was set to 15,000 Da/s. All data were collected and processed using MS Workstation/Data Review software (v6.9.1, Varian). Quality control (QC) was ensured by injecting a pooled QC sample after every 10 experimental runs to monitor instrumental stability. Solvent blanks (methanol–water) were analyzed to account for background contamination. Real-time mass calibration was performed using leucine enkephalin (m/z 556.2771) as the lock mass standard.

### Data analysis

#### Metabolite annotation and confidence levels

The raw mass spectral files were first converted to mzXML format via ProteoWizard’s MSConvert tool (v3.0.8789) [23]. Peak detection, filtration, and alignment were performed using the XCMS package (v3.12.0) on the R platform [24]. Specifically, feature detection used the centWave algorithm, with subsequent filtration (signal-to-noise ratio > 10) and retention time alignment to produce a preliminary feature table. After signal correction via QC samples, a normalized quantitative matrix was obtained. The entire workflow incorporated verification checkpoints in R (v4.1.0) for reproducibility. Metabolite annotation was performed by querying public spectral databases, including MassBank [25], LipidMaps [26], mzCloud [27], and KEGG [28]. Final metabolite annotation was conducted by matching against public databases with a mass accuracy of < 30 ppm. Annotation confidence levels were assigned according to the Metabolomics Standards Initiative (MSI) guidelines. The majority of identifications reported here are at Level 2 (putatively annotated compounds), based on matching to library MS/MS spectra. Detailed annotation information for key differential metabolites, including database match scores and spectral similarity, is provided in Table S3.

#### Metabolomic data analysis

Statistical analyses were performed separately on datasets acquired in positive and negative ionization modes. Multivariate statistical analyses, including principal component analysis (PCA), partial least squares-discriminant analysis (PLS-DA), orthogonal PLS-DA (OPLS-DA), and permutation testing (*n* = 200), were conducted using the R package *Ropls* (v1.6.2). Differential metabolites were identified by integrating univariate and multivariate results from each mode, and the combined list was used for subsequent functional analysis. Specifically, candidates were required to simultaneously satisfy *p* < 0.05 (adjusted by False Discovery Rate, FDR), an OPLS-DA-derived VIP score > 1, and an absolute fold change |FC| > 1.2. Compounds fulfilling all criteria were considered potential biomarkers and subsequently subjected to KEGG pathway enrichment analysis for functional interpretation. All data were preprocessed with log₂ transformation and Pareto scaling prior to statistical modeling.

#### Pathway enrichment analysis

Metabolic pathway enrichment analysis was performed based on a hypergeometric test probability model [29]. Significantly enriched pathways were visualized using the KEGG Mapper toolset to map and interrogate the relationships between differential exudates and biochemical pathways.

#### Analysis of variance (ANOVA)

A three-way analysis of variance (ANOVA) was performed on the root morphological data using SPSS software (Version 27, IBM, U.S.A.) to evaluate the main effects of cultivar, light intensity, P supply, and their interactions. In cases where significant effects were identified (*p* < 0.05), Duncan’s multiple range test was conducted for post-hoc comparison among group means. Results are expressed as mean ± standard error (Mean ± SE), and significant differences (*p* < 0.05) are designated with different lowercase letters in graphical representations.

## Results

### Effects of light intensity and P supply on rice root morphology and plant P content

**Fig. 1:**
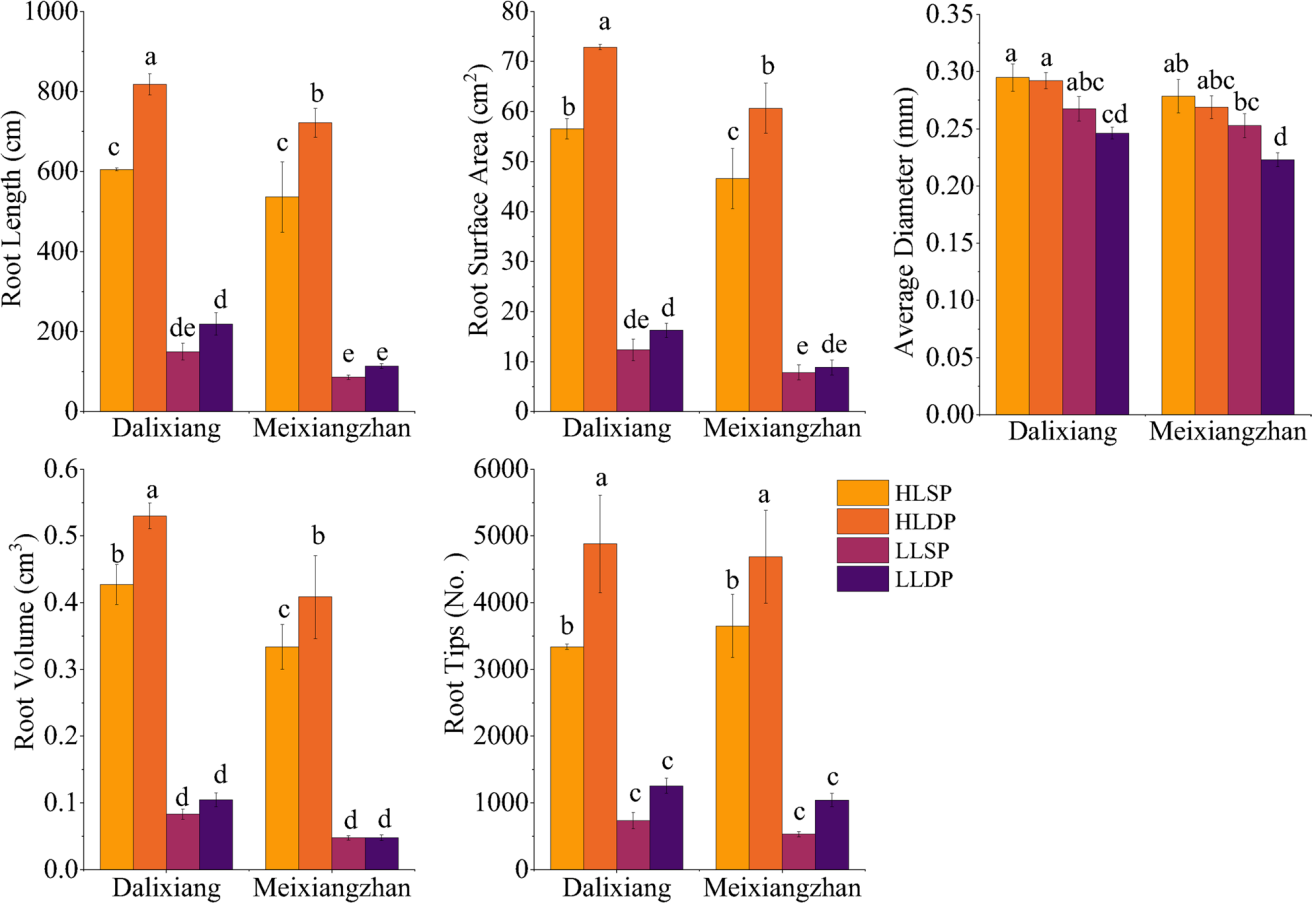
Variation in root morphology of Dalixiang and Meixiangzhan under different light intensity and P supply at the five-leaf stage (*n* = 3). Vertical bars represent standard error of the mean. Bars labeled with different lowercase letters indicate statistically significant differences (*p* < 0.05) between treatments, *n* = 3. HLSP refers to high light with sufficient P, HLDP refers to high light with deficient P, LLSP refers to low light with sufficient P, and LLDP refers to low light with deficient P

Morphological characterization under controlled conditions confirmed the contrasting P efficiency of the two cultivars. Compared to low light intensity, both cultivars exhibited significant increases in root length, surface area, root diameter, root volume, and tips number under high light intensity. Under high light, P deficiency significantly enhanced root length, surface area, volume, and tips number in both cultivars (Fig. 1). In contrast, P deficiency had no effect on root length, surface area, volume, or number under low light intensity for either cultivar, but significantly reduced the average root diameter of Meixiangzhan. Under high light with P deficiency (HLDP), Dalixiang exhibited superior root morphological plasticity (Fig. 1), whereas Meixiangzhan showed a minimal response.

Compared to the P-susceptible cultivar, the low-P-tolerant cultivar Dalixiang exhibited higher photosynthetic efficiency (Fig. S2). While P supply had no significant effect on root-to-shoot ratio for either cultivar under low light intensity (Table 1). Cultivar difference was observed, with Dalixiang exhibiting superior root growth performance compared to Meixiangzhan. Under the same light, P supply did not affect root-to-shoot ratio in Meixiangzhan. However, P deficiency led to enhanced root growth and a significant 121.4% increase in root-to-shoot ratio in Dalixiang under high light.

**Table 1:** Effects of varying light intensity and P supply on root/shoot ratio and P content in rice at the five-leaf stage(*n* = 3)。

Cultivar	Treatment	*R*/S ratio	Shoot *P* content (g/kg DW)	Root *P* content (g/kg DW)
Dalixiang	HLSP	0.14 ± 0.02d	2.58 ± 0.01f	3.30 ± 0.05f
	HLDP	0.31 ± 0.02a	1.39 ± 0.01 h	2.78 ± 0.03 g
	LLSP	0.16 ± 0.01 cd	2.93 ± 0.01e	6.19 ± 0.16a
	LLDP	0.16 ± 0.00 cd	1.53 ± 0.01 g	4.87 ± 0.03c
Meixiangzhan	HLSP	0.20 ± 0.02bc	4.49 ± 0.02b	5.27 ± 0.02b
	HLDP	0.22 ± 0.02b	3.46 ± 0.02d	3.40 ± 0.04f
	LLSP	0.16 ± 0.01 cd	4.22 ± 0.01c	4.44 ± 0.05d
	LLDP	0.19 ± 0.02bc	4.81 ± 0.03a	3.92 ± 0.03e
Three-way ANOVA				
Cultivar (C)		0.002ns	50466.53**	0.178
Light(L)		23.71**	1675.98**	982.98**
Phosphorus(P)		31.82**	6336.12**	855.06**
C×L		3.20ns	225.71**	1266.16**
C×P		10.61*	3163.58**	10.51**
L×P		15.54**	1379.11**	11.18**
C×L×P		18.30**	2304.08**	227.67**

Results are presented as mean ± standard error (SE). Different lowercase letters indicate significant differences(*p* < 0.05)among light intensity and P treatments

Asterisks denote significant differences between specific treatment comparisons: *p < 0.05, **p < 0.01; ns, not significant (*p* ≥ 0.05)

HLSP refers to high light with sufficient P, HLDP refers to high light with deficient P, LLSP refers to low light with sufficient P, and LLDP refers to low light with deficient P

### Quality control assessment

**Fig. 2:**
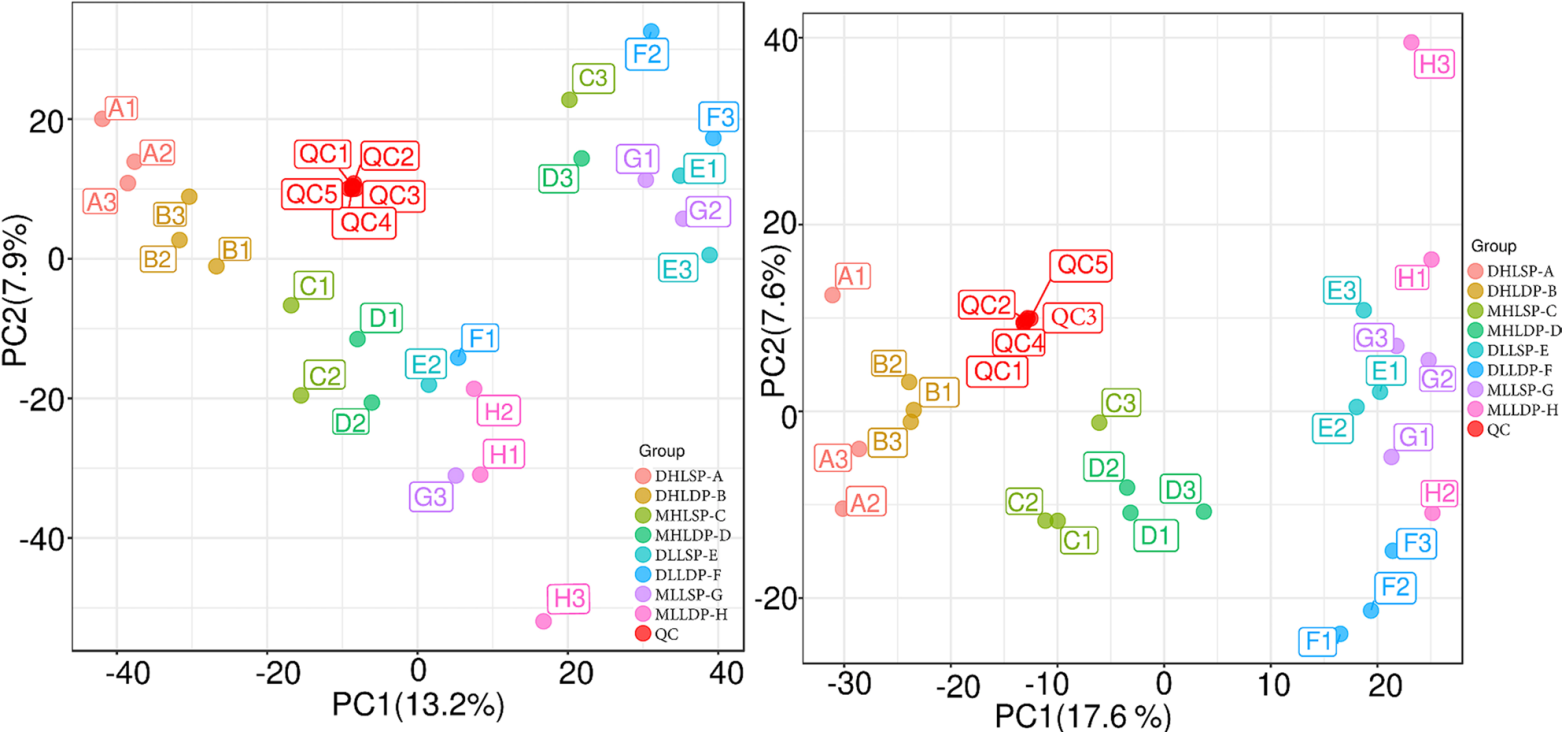
PCA score plots of QC samples in positive (**a**) and negative (**b**) ion modes. Red points represent QC samples; points in other colors represent experimental samples

QC (quality control) samples (QC1-QC4) showed tight clustering in both analytical modes, clearly separated from the treatment groups. The technical reproducibility of QC samples (RSD < 15%) in both analytical modes confirmed excellent analytical stability throughout the experimental process (Fig. 2). Substantial separation between treatment groups was observed along both PC1 and PC2, accounting for 13.2% and 7.9% of total variance in positive ion mode, 17.6% and 7.6% in negative ion mode, respectively. The distinct clustering patterns in PCA score plots demonstrate significant effects of light intensity and P treatments on rice root exudation profiles.

### KEGG pathway enrichment analysis of differential root exudates

**Fig. 3:**
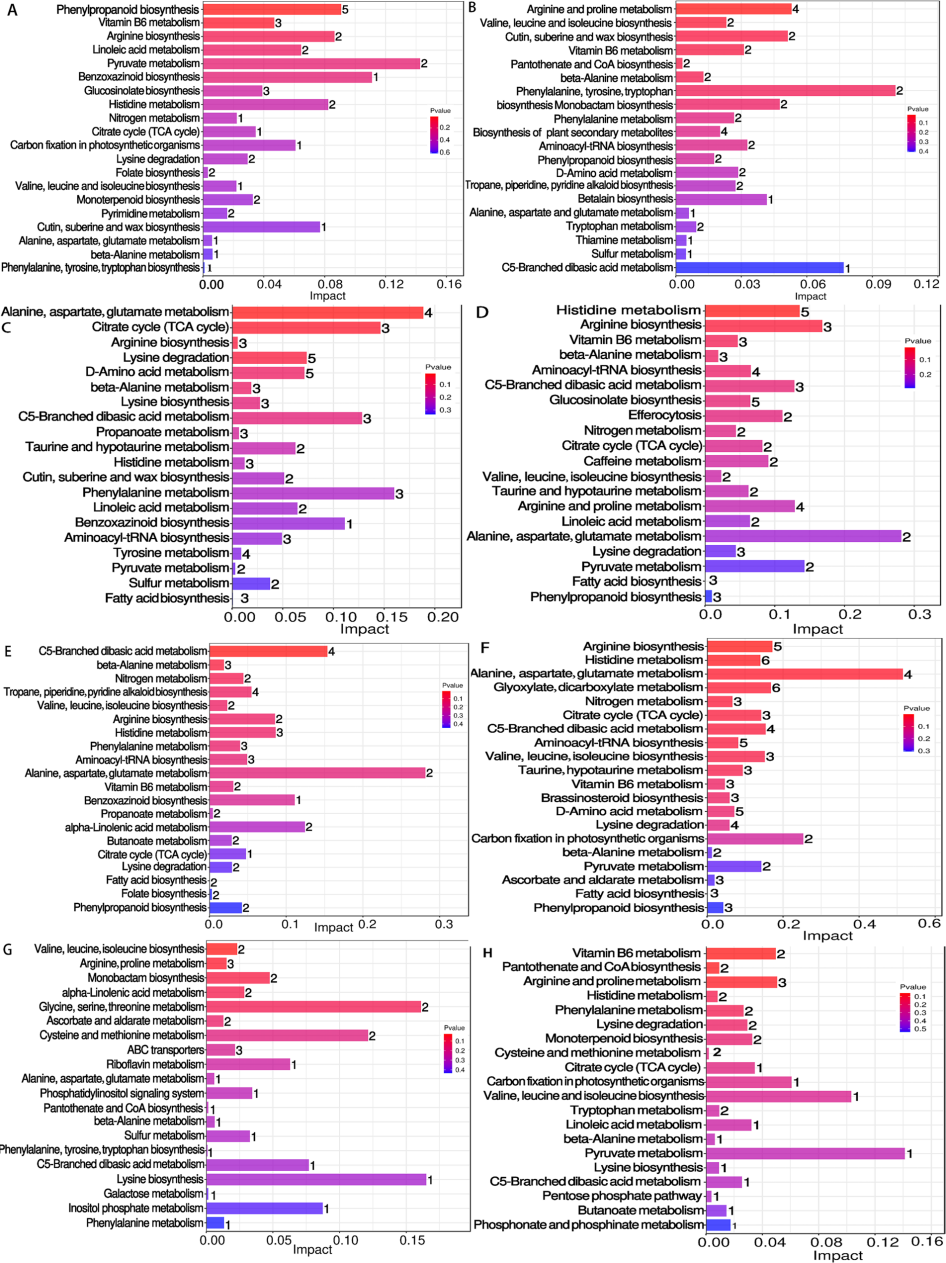
Enrichment analysis of differentially expressed root exudates in the low-P-tolerant rice cultivar Dalixiang (D) under high light with sufficient P (HLSP), high light with P deficiency (HLDP), low light with sufficient P (LLSP), and low light with P deficiency (LLDP) treatments at the five-leaf stage. (**A** DHLSP vs DHLDP, **B** DLLSP vs DLLDP, **C** DHLSP vs DLLSP, **D** DHLDP vs DLLDP). Enrichment analysis of differentially expressed root exudates between the low-P-tolerant cultivar Dalixiang (D) and low-P-susceptible cultivar Meixiangzhan (M) at the five-leaf stage. (**E** DHLSP vs MHLSP, **F** DHLDP vs MHLDP, **G** DLLSP vs MLLSP, **H** DLLDP vs MLLDP)

KEGG pathway enrichment analysis (Metabolite Set Mass Spectrometry, MS/MS) on differential root exudates from Dalixiang (Fig. 3) revealed distinct metabolic pathway enrichments (*p* < 0.1). For HLSP vs. HLDP, 2 pathways showed significant enrichment as phenylpropanoid biosynthesis and vitamin B6 metabolism. For LLSP vs. LLDP, 6 pathways were significantly enriched, namely arginine and proline metabolism, valine, leucine and isoleucine biosynthesis, cutin, suberine and wax biosynthesis, vitamin B6 metabolism, pantothenate and CoA biosynthesis as well as beta-alanine metabolism. For HLSP vs. LLDP, 4 pathways demonstrated significant enrichment as alanine, aspartate and glutamate metabolism, citrate cycle (TCA cycle), arginine biosynthesis and Lysine degradation. For HLDP vs. LLDP, 2 pathways were significantly enriched as histidine metabolism and arginine biosynthesis. This indicates that light intensity primarily modulates carbon and nitrogen flow into exudates, whereas P supply specifically reprograms stress-responsive and biosynthetic pathways.

Cultivar differences in root exudates between the Dalixiang (D) and Meixiangzhan (M) rice were performed in KEGG pathway enrichment analysis (Fig. 3). Under HLSP, 2 metabolic pathways showed relatively high enrichment (*p* < 0.1) in the comparison between cultivars: C5-branched dibasic acid metabolism and beta-alanine metabolism. Under HLDP, 10 metabolic pathways were significantly enriched in the cultivar comparison, including arginine biosynthesis, histidine metabolism, alanine, aspartate and glutamate metabolism, glyoxylate and dicarboxylate metabolism, nitrogen metabolism, citrate cycle (TCA cycle), C5-branched dibasic acid metabolism, aminoacyl-tRNA biosynthesis, valine, leucine and isoleucine biosynthesis, and taurine and hypotaurine metabolism. Under LLSP, 5 metabolic pathways were notably enriched between cultivars, including valine, leucine and isoleucine biosynthesis, arginine and proline metabolism, monobactam biosynthesis, alpha-linolenic acid metabolism, and glycine, serine and threonine metabolism. Under LLDP, 3 metabolic pathways exhibited significant enrichment, including vitamin B6 metabolism, pantothenate and CoA biosynthesis, and arginine and proline metabolism.

Under high light, the number of metabolic pathways responding to P deficiency significantly increased in the cultivar comparison (Fig. 3). In contrast, under low light stress, both the number of responsive metabolic pathways and metabolites decreased. The effects of light intensity and P supply on root exudates are not mediated through a single pathway but involve interactive mechanisms across multiple pathways, encompassing plant carbon assimilation, nitrogen metabolism, and synthesis of secondary metabolites as part of adaptive responses.

### Identification and analysis of differentially exuded metabolites in root exudations

Light intensity and P supply significantly altered rice root exudation profile (Table 2). Under high light, alterations in P availability induced 117 differentially exuded metabolites (74 up-regulated/43 down-regulated). Under low light, P supply resulted in 68 differential metabolites (41 up-regulated/27 down-regulated). Under sufficient P, shifts in light intensity led to changes in 155 metabolites (113 up-regulated/42 down-regulated), while under P deficiency, variations in light intensity triggered responses in 146 metabolites (106 up-regulated/40 down-regulated). The total number of differential metabolites induced by light intensity (DHLSP vs. DLLSP + DHLDP vs. DLLDP = 301) was significantly higher than that induced by P supply (DHLSP vs. DHLDP + DLLSP vs. DLLDP = 185) (Table 2, *p* < 0.05).

Under high light, sufficient P supply induced 113 differentially metabolites between cultivars (89 up-regulated/24 down-regulated) (Table 2), while P deficiency led to 158 differential metabolites (104 up-regulated/54 down-regulated). Under low light with sufficient P, 54 differential metabolites were identified (31 up-regulated/23 down-regulated), while P deficiency resulted in 56 differential metabolites (24 up-regulated/32 down-regulated). The response of cultivar differences in root exudation to P deficiency (DHLDP vs. MHLDP + DLLDP vs. MLLDP = 214 metabolites) was more pronounced than P-sufficient conditions (DHLSP vs. MHLSP + DLLSP vs. MLLSP = 167 metabolites). Across all light and P treatments, Dalixiang produced a greater number of differentially exudated metabolites than Meixiangzhan (Table 2; Table S2).

**Table 2:** Differential root exudates of the low-P-tolerant rice cultivar Dalixiang (D) under high light with P deficiency (HLDP), low light with sufficient P (LLSP), and low light with P deficiency (LLDP) treatments, and its comparative analysis with the cultivar Meixiangzhan (M) (*p* < 0.05) at the five-leaf stage

Comparison	Up	Down	Total DE
DHLSP vs. DHLDP	74	43	117
DLLSP vs. DLLDP	41	27	68
DHLSP vs. DLLSP	113	42	155
DHLDP vs. DLLDP	106	40	146
DHLSP vs. MHLSP	89	24	113
DHLDP vs. MHLDP	104	54	158
DLLSP vs. MLLSP	31	23	54
DLLDP vs. MLLDP	24	32	56

**Fig. 4:**
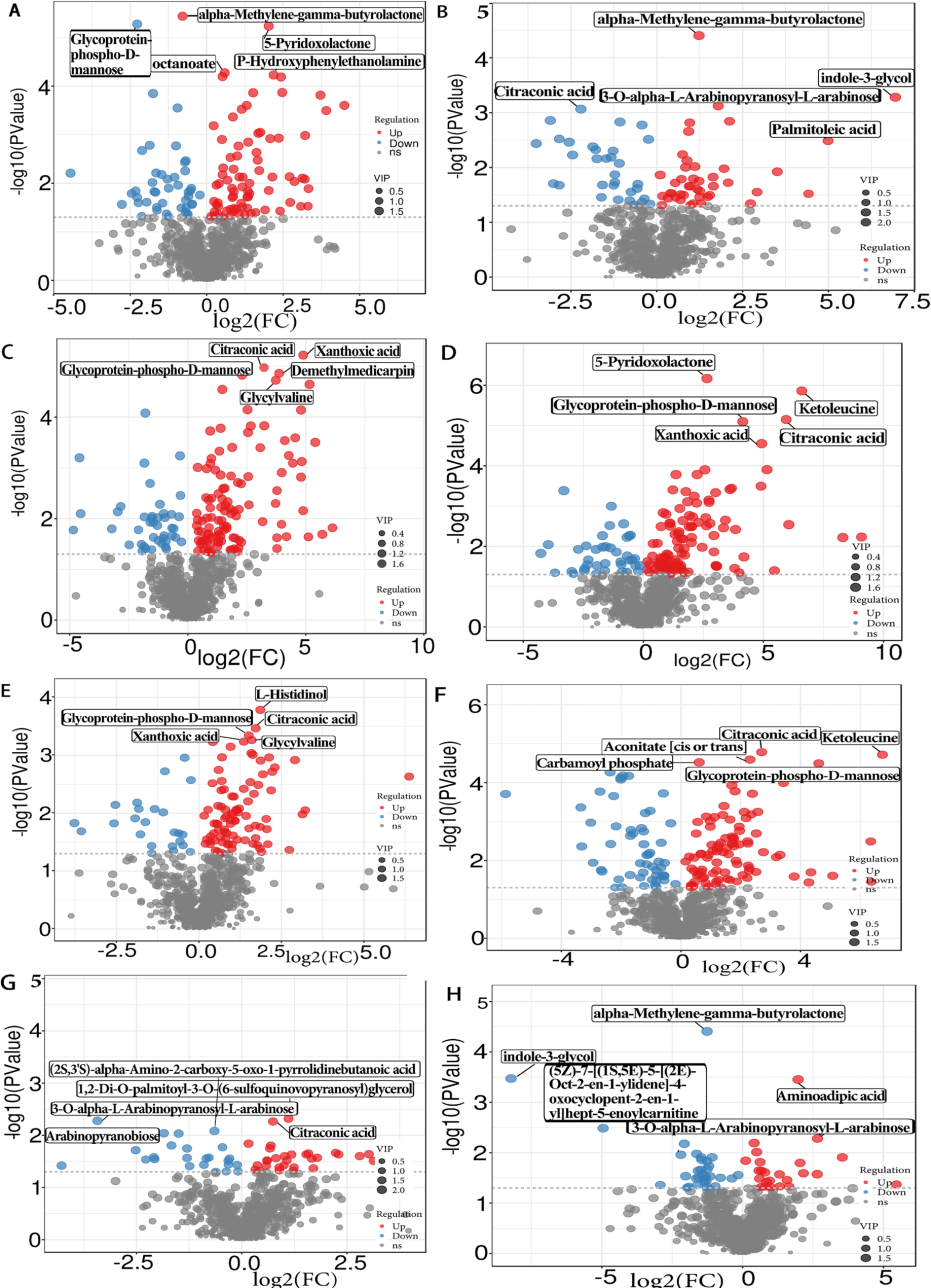
Volcano plots illustrating differentially abundant root exudates of the low-P-tolerant rice cultivar Dalixiang (D) under high light with sufficient P (HLSP), high light with P deficiency (HLDP), low light with sufficient P (LLSP), and low light with P deficiency (LLDP) treatments at the five-leaf stage. (**A** DHLSP vs. DHLDP, **B** DLLSP vs. DLLDP, **C **DHLSP vs. DLLSP, **D** DHLDP vs. DLLDP). Metabolites with significant up-regulation are shown in red, those with significant down-regulation in blue, and non-significant metabolites in gray. Volcano plots of differentially abundant root exudates between low-P-tolerant cultivar Dalixiang (D) and low-P-susceptible cultivar Meixiangzhan (M) at the five-leaf stage. (**E** DHLSP vs. MHLSP, **F** DHLDP vs. MHLDP, **G** DLLSP vs. MLLSP, **H** DLLDP vs. MLLDP). Metabolites with significant up-regulation are shown in red, those with significant down-regulation in blue, and non-significant metabolites in gray

Under high light, P supply induced notable changes in exudates of Dalixiang (Fig. 4, Table 3), including alpha-methylene-gamma-butyrolactone, glycoprotein-phospho-D-mannose, 5-pyridoxolactone, octanoate, p-hydroxyphenylethanolamine, and carbamoyl phosphate. under low light intensity, alterations in P availability led to differential exudation of alpha-methylene-gamma-butyrolactone, indole-3-glycol, 3-O-alpha-L-arabinopyranosyl-L-arabinose, citraconic acid, and palmitoleic acid. Under sufficient P supply, light intensity shifts triggered changes in xanthoxic acid, citraconic acid, demethylmedicarpin, glycoprotein-phospho-D-mannose, and glycylvaline. Under P deficiency, variations in light intensity resulted in differential accumulation of 5-pyridoxolactone, ketoleucine, citraconic acid, glycoprotein-phospho-D-mannose, and xanthoxic acid. Key differentially expressed metabolites responded to light (DHLSP vs. DLLSP, DHLDP vs. DLLDP) exhibited broader fold change magnitude (log_2_FC) than P (DHLSP vs. DHLDP, DLLSP vs. DLLDP). For example, the response of citraconic acid to P deficiency was substantially greater under high light (62-fold) than under low light (3.4-fold).

For differential exudates between the cultivars (Fig. 4, Table 4), HLSP induced L-histidinol, citraconic acid, glycoprotein-phospho-D-mannose, glycylvaline, and xanthoxic acid. HLDP identified citraconic acid, ketoleucine, aconitate, carbamoyl phosphate, and glycoprotein-phospho-D-mannose. Under LLSP, the cultivar differentiated exudates consisted of 1,2-di-O-palmitoyl-3-O-(6-sulfoquinovopyranosyl) glycerol, 3-O-alpha-L-arabinopyranosyl-L-arabinose, citraconic acid, (2 S,3’S)-alpha-amino-2-carboxy-5-oxo-1-pyrrolidinebutanoic acid, and arabinopyranobiose. LLDP induced alpha-methylene-gamma-butyrolactone, indole-3-glycol, aminoadipic acid, (5Z)-7-[(1 S,5E)-5-[(2E)-oct-2-en-1-ylidene]-4-oxocyclopent-2-en-1-yl]hept-5-enoylcarnitine, and 3-O-alpha-L-arabinopyranosyl-L-arabinose.

**Table 3:** Key differentially expressed metabolites in the low-P-tolerant rice cultivar Dalixiang (D) under high light with sufficient P (HLSP), high light with P deficiency (HLDP), low light with sufficient P (LLSP), and low light with P deficiency (LLDP) treatments at the five-leaf stage

Comparison	Compound Name	mzmed	rtmed	log_2_FC	VIP	Up/ Down
DHLSP vs. DHLDP	alpha-Methylene-gamma-butyrolactone	116.1	308.0	-0.96	1.83	↓
	Glycoprotein-phospho-D-mannose	179.1	379.7	-2.27	1.79	↓
	5-Pyridoxolactone	164.0	100.1	2.03	1.79	↑
	octanoate	166.1	413.6	0.59	1.82	↑
	P-Hydroxyphenylethanolamine	154.1	151.1	2.19	1.82	↑
	Carbamoyl phosphate	159.0	41.2	0.52	1.82	↑
DLLSP vs. DLLDP	alpha-Methylene-gamma-butyrolactone	116.1	308.0	1.23	2.06	↑
	indole-3-glycol	161.1	48.9	6.95	2.03	↑
	3-O-alpha-L-Arabinopyranosyl-L-arabinose	300.2	408.4	-3.37	2.03	↓
	Citraconic acid	129.1	320.7	1.79	1.83	↑
	Palmitoleic acid	253.2	427.2	4.99	1.78	↑
DHLSP vs. DLLSP	Xanthoxic acid	265.1	299.8	4.89	1.58	↑
	Citraconic acid	129.1	320.7	3.68	1.58	↑
	Demethylmedicarpin	255.2	344.4	3.87	1.58	↑
	Glycoprotein-phospho-D-mannose	179.1	379.7	2.30	1.58	↑
	Glycylvaline	173.1	52.9	3.73	1.58	↑
DHLDP vs. DLLDP	5-Pyridoxolactone	164.0	100.1	2.64	1.58	↑
	Ketoleucine	129.1	423.5	6.6	1.58	↑
	Citraconic acid	129.1	320.7	5.95	1.58	↑
	Glycoprotein-phospho-D-mannose	179.1	379.7	4.14	1.58	↑
	Xanthoxic acid	265.1	299.8	4.54	1.58	↑

The log₂ fold change (log₂FC) is calculated as (first group) / (second group) for each comparison listed in the “Comparison” column

A positive value indicates higher abundance in the first group; a negative value indicates higher abundance in the second group

**Table 4:** Key differentially expressed root exudates between Dalixiang (D) and Meixiangzhan (M) cultivars under high light with sufficient P (HLSP), high light with P deficiency (HLDP), low light with sufficient P (LLSP), and low light with P deficiency (LLDP) treatments at the five-leaf stage

Comparison	Compound Name	mzmed	rtmed	log_2_FC	VIP	Up/ Down
DHLSP vs. MHLSP	L-Histidinol	124.1	412.7	1.86	1.80	↑
	Citraconic acid	129.1	320.7	1.57	1.71	↑
	Glycoprotein-phospho-D-mannose	179.1	379.7	1.51	1.71	↑
	Glycylvaline	173.1	52.9	1.6	1.71	↑
	Xanthoxic acid	265.15	299.8	1.36	1.71	↑
DHLDP vs. MHLDP	Citraconic acid	129.1	320.7	2.70	1.71	↑
	Ketoleucine	129.1	423.5	6.74	1.71	↑
	Aconitate [cis or trans]	173.0	43.3	2.31	1.71	↑
	Carbamoyl phosphate	159.0	41.2	0.61	1.80	↑
	Glycoprotein-phospho-D-mannose	179.1	379.7	4.61	1.71	↑
DLLSP vs. MLLSP	1,2-Di-O-palmitoyl-3-O-(6-sulfoquinovopyranosyl) glycerol	793.5	413.9	1.11	1.86	↑
	3-O-alpha-L-Arabinopyranosyl-L-arabinose	300.2	408.4	-3.43	2.00	↓
	Citraconic acid	129.1	320.7	0.74	1.85	↑
	(2 S,3’S)-alpha-Amino-2-carboxy-5-oxo-1-pyrrolidinebutanoic acid	214.0	67.6	-0.65	1.97	↓
	Arabinopyranobiose	300.2	382.4	-1.85	1.96	↓
DLLDP vs. MLLDP	alpha-Methylene-gamma-butyrolactone	116.1	308.0	-1.26	1.91	↓
	indole-3-glycol	161.1	48.9	-8.22	1.89	↓
	Aminoadipic acid	118.1	297.7	1.97	1.89	↑
	(5Z)-7-[(1 S,5E)-5-[(2E)-Oct-2-en-1-ylidene]-4-oxocyclopent-2-en-1-yl] hept-5-enoylcarnitine	443.3	248.6	-4.95	1.83	↓
	3-O-alpha-L-Arabinopyranosyl-L-arabinose	300.2	408.4	2.66	1.81	↑

The log₂ fold change (log₂FC) is calculated as (first group) / (second group) for each comparison listed in the “Comparison” column

A positive value indicates higher abundance in the first group; a negative value indicates higher abundance in the second group

### Effects of exogenous differential metabolites on growth and P uptake in P-susceptible rice Meixiangzhan

Based on the differentially expressed metabolites identified above, seven compounds characterized by low cost, significant expression levels, and high detection frequency were selected to evaluate their efficacy in alleviating P deficiency in the P-susceptible rice cultivar Meixiangzhan (Table 5). The results demonstrated that alpha-methylene-gamma-butyrolactone significantly increased both shoot and root dry weight under P deficiency, exhibiting the most pronounced effect. Citraconic acid, xanthoxic acid, and indole-3-glycol also showed considerable positive effects with higher shoot and root P uptake. Compared to CK, Glycoprotein-phospho-D-mannose and Ketoleucine decreased shoot and root P content, especially for a reduction in P uptake but increase in shoot dry weight of Glycoprotein-phospho-D-mannose. In contrast, L-histidinol did not promote plant growth, but increased P uptake in shoot.

**Table 5:** Effects of exogenous application of selected differential metabolites on dry weight, P content and uptake in the P-susceptible rice cultivar Meixiangzhan under high light with P deficiency at the five-leaf stage

Exudates	Shoot dry weight (g pot^− 1^)	Shoot *P* content (mg g^− 1^)	Shoot *P* uptake (mg pot^− 1^)	Root dry weight (g pot^− 1^)	Root *P* content (mg g^− 1^)	Root *P* uptake (mg pot^− 1^)
CK	2.92 ± 0.07c	3.46 ± 0.02b	9.9 ± 0.59c	0.63 ± 0.02 cd	3.40 ± 0.04ab	2.16 ± 0.29c
+ Citraconic acid	3.70 ± 0.33bc	3.73 ± 0.21b	13.8 ± 1.49b	0.92 ± 0.09b	3.18 ± 0.21bc	2.94 ± 0.35b
+Xanthoxic acid	3.80 ± 0.22b	4.54 ± 0.05a	17.2 ± 1.19a	0.87 ± 0.05b	3.40 ± 0.07ab	2.97 ± 0.20b
+ L-Histidinol	2.96 ± 0.26c	4.62 ± 0.27a	13.7 ± 1.71b	0.55 ± 0.05d	3.81 ± 0.33ab	2.11 ± 0.30c
+indole-3-glycol	3.54 ± 0.16bc	4.25 ± 0.01a	15.1 ± 0.71b	0.92 ± 0.03b	3.97 ± 0.03a	3.63 ± 0.12a
+alpha-Methylene-gamma-butyrolactone	4.80 ± 0.50a	1.66 ± 0.12c	8.0 ± 0.24d	1.33 ± 0.11a	2.64 ± 0.14 cd	2.98 ± 0.12b
+ Glycoprotein-phospho-D-mannose	3.91 ± 0.03b	0.79 ± 0.07d	3.1 ± 0.26f	0.80 ± 0.03bc	2.22 ± 0.08d	1.78 ± 0.13d
+Ketoleucine	3.52 ± 0.31bc	1.36 ± 0.24c	4.8 ± 0.53e	0.69 ± 0.07 cd	3.27 ± 0.29bc	2.25 ± 0.24c

The results are presented as mean ± SE. Different lowercase letters indicate significant differences (*p* < 0.05) among treatments with exogenous addition of different secretory metabolites

## Discussion

### Synergistic effects of light intensity and P availability on rice growth and P content

Under sufficient P supply, plants typically prioritize the allocation of photo-assimilates to shoots, whereas P deficiency promotes greater partitioning of carbohydrates to the roots, resulting in reduced shoot growth and an increased root-to-shoot ratio [30]. Morphologically, plants enhance root morphology by promoting primary root growth [31], stimulating lateral root formation [32], and elongating root hairs [33] to expand the root absorption surface. Physiologically, certain species primarily employ chemical strategies such as exudation of organic compounds [34] to improve soil P availability, indicating divergent P efficiency mechanisms among plant species. Root development and morphology often differ between hydroponic and soil systems due to differences in physical resistance and nutrient distribution. While the rock-P method provides a slow-release P source, it does not simulate the heterogeneous and spatially variable P pools found in soil, such as adsorbed P, occluded P, and organic P forms.

Under high light and P deficiency, the low-P-tolerant Dalixiang significantly enhanced root growth and increased its root-to-shoot ratio by 121.4% (Table 1), indicating a carbon partitioning priority to roots as a potential P tolerance mechanism [35]. In contrast, the root-to-shoot ratio of the P-susceptible cultivar Meixiangzhan was significantly affected by P availability (Table 1), indicating a limited plasticity in carbon partitioning to roots and thus a reduced adaptability to P deficiency [36]. Under low light, root morphology was unaffected by P availability in both cultivars (Table 1; Fig. 1). Under high light, P deficiency significantly enhanced root length, surface area, and tip number, particularly in the low-P-tolerant Dalixiang. The superior root growth in Dalixiang was consistent with its concurrently higher photosynthetic efficiency and greater number of green leaves (Fig. S1), indicating a coordinated response. Although Dalixiang showed better overall growth under high light (Table 1), its tissue P content remained unchanged, potentially due to a growth dilution effect.

### Root exudates in response to light intensity and P supply in contrasting cultivars

P deficiency typically enhances root exudation rates in plants, with the total quantity of differential metabolites being determined by both exudation rate and metabolic regulation [37, 38]. For low-P-tolerant cultivar Dalixiang, variations in light intensity (HL vs. LL) induced a greater number of differential metabolites compared to P supply (SP vs. DP) (301 vs. 185), suggesting that photo-assimilates may directly regulate root exudation [39]. However, the biological significance of these metabolic shifts extends beyond simple numerical comparisons. When examining the magnitude of changes (log₂FC), several key metabolites exhibited substantially stronger responses under specific treatment combinations. For instance, in comparison DHLDP vs. DLLDP, metabolites such as ketoleucine (log₂FC = 6.60, ~ 97-fold) and citraconic acid (log₂FC = 5.95, ~ 62-fold) showed extreme upregulation, whereas in DLLSP vs. DLLDP, citraconic acid exhibited a much weaker response (log₂FC = 1.79, ~ 3.4-fold; Table 3). This indicates that high light not only induces a greater number of differential metabolites but also significantly amplifies the response intensity of specific metabolites to P deficiency.

Furthermore, distinct functional classes of metabolites were preferentially influenced by each factor (Fig. 3). P deficiency particularly upregulates pathways involved in amino acid metabolism and stress-associated biosynthesis (e.g., vitamin B6, pantothenate, beta-alanine), which may contribute to cellular homeostasis, signaling, or microbial recruitment under nutrient limitation [40]. Conversely, variations in light intensity predominantly alter pathways linked to photosynthate allocation and central energy metabolism, such as the TCA cycle and amino acid biosynthesis/ degradation [41]. This suggests that light acts as a master regulator of carbon supply for exudation, while P status fine-tunes the metabolic composition towards specific adaptive functions.

The low-P-tolerant cultivar Dalixiang consistently exhibited a greater abundance of differential metabolites compared to Meixiangzhan across all treatments (Table 2, Table S2). This difference was particularly pronounced under P deficiency, where the number of cultivar-specific differential metabolites (214) significantly exceeded that under P-sufficient conditions (167). More importantly, the functional categories of these metabolites differed substantially between cultivars under P deficiency. The superior plasticity of Dalixiang lies in its ability to activate a broader suite of metabolic pathways under high light and P stress, particularly those linking carbon, nitrogen, and energy metabolism (Fig. 3, Table S2). While Meixiangzhan showed limited metabolic pathways to cope with multiple stressors, consistent with previous reports indicating constrained root plasticity in P-susceptible cultivars [42].

The attenuated root and exudate responses to P deficiency under low light can be attributed to photosynthetic carbon limitation [43]. Under such carbon-limited conditions, plants may prioritize essential maintenance functions over facultative adaptive responses such as enhanced root proliferation or exudation of carbon-rich compounds in response to P deficiency [44]. This explained why the number of enriched pathways decreased markedly under low light conditions (Fig. 3). This interpretation is further supported by the carbon allocation patterns inferred from increased root-to-shoot ratio as affected by P deficiency under high light but no effect under low light (Table 1). The energetic cost of mounting a full adaptive response (e.g., root growth, exudation) appears to exceed the available photoassimilate supply under low light, thereby masking the cultivar-specific P-stress responses observed under high light. Thus, light availability determines whether genetic potential for P acquisition is expressed, indicating that agronomic evaluations of nutrient-use efficiency must consider light availability, as it can be a decisive factor in whether intrinsic tolerance traits are fully expressed.

### Functional test of differential exudates in alleviating P deficiency

Previous studies have primarily focused on the light-responsive characteristics of specific compounds—such as citrate [45], phytosiderophores [46], and catechins [47]—with some even identifying diurnal rhythms [48] in root exudation. Within the metabolic pathways associated with differential exudates, amino acid metabolism and related metabolites were also significantly regulated by both light intensity and P supply (Fig. 3). Notably, compounds such as arginine, proline, and alanine, aspartate, and glutamate may be directly involved in plant responses to stress [49]. Low light and low P are stresses that could potentially cause cellular damage [30], but MDA content indicates that the release is specific, not a result of generalized damage (Fig. S3). For example, LLSP vs. LLDP exudated the most amino-acid (Fig. 3) while did not show the highest MDA content in leaf and root (Fig. S3). We assume that, on the one hand, the exudation of amino-acid may be an active process where amino acids act as signals to recruit phosphate-solubilizing microbes, which is related to higher root acid phosphatase activity [18]. On the other hand, amino acids influence plant adaptability by modulating metabolic homeostasis and signal transduction [50].

Exogenous application assays among the seven metabolites were tested on P-susceptible Meixiangzhan and showed diverse effects (Table 5, Fig. S2). The pronounced growth promotion by alpha-methylene-gamma-butyrolactone suggests a decoupling of growth promotion from P acquisition. Its primary effect may involve stimulation of root growth or alterations in carbon allocation rather than direct enhancement of P mobilization, potentially acting as a signaling molecule that promotes root proliferation [51]. Similarly, Glycoprotein-phospho-D-mannose and Ketoleucine increased plant dry weight but decreased shoot P content and total P uptake (Table 5). Glycoconjugates like Glycoprotein-phospho-D-mannose may serve as microbial attractants or substrates, but it did not improve P availability [52].

Interestingly, citraconic acid and xanthoxic acid, both upregulated under high light and P deficiency, significantly increased shoot P content and total P uptake (Table 5), suggesting they enhance P mobilization, likely through chelation or rhizosphere acidification [7, 53]. Citraconic acid, as a citric acid derivative, may operate via proton-coupled transport mechanisms to lower rhizospheric pH and displace P from Fe/Al complexes [54]. L-histidinol did not significantly improve plant growth but increased shoot P content and maintained total P uptake, suggesting it might contribute to cellular homeostasis or facilitate P remobilization without allocating additional carbon to growth [49, 55]. The effectiveness of these compounds in complex soil environments, where microbial activity and soil chemistry modulate their effectiveness, remains a critical subject for future field studies.

## Conclusion

This study provides novel evidence that light intensity overrides P supply as the dominant environmental factor reshaping the root exudation profile in rice, based on both the number and magnitude of metabolic changes. Several previously unreported metabolites co-regulated by light and P were identified to expand understanding of root-mediated P acquisition. Carbon limitation under low light suppresses the metabolic flexibility of P-tolerant cultivars, directly linking photosynthetic capacity to rhizosphere function. These insights provide a theoretical basis for improving P utilization efficiency in rice production.

## Supplementary Information

Supplementary Material 1.

## Data Availability

The data or material of this study are available from the first author, G.L. upon reasonable request.

## Abbreviations

HLSP: High light with sufficient P.
HLDP: High light with deficient P.
LLSP: Low light with sufficient P.
LLDP: Low light with deficient P.
