# Supplementary material for: Metabolomic profiling reveals root exudation pattern as regulated by phosphorus and light in contrasting rice cultivars
Source: BMC Plant Biol. 2026 Mar 26;26:740. doi: 10.1186/s12870-026-08527-5 (PMC13104452; doi:10.1186/s12870-026-08527-5)
Supplement: Supplementary file 1 — Supplementary Material 1. [file 12870_2026_8527_MOESM1_ESM.docx]

Supplementary figure. 1 maximum photosystem II quantum yield (PhiPS2) of two rice cultivars under different light intensities and P supplies.

Vertical bars represent standard error of the mean. Bars labeled with different lowercase letters indicate statistically significant differences (*p*<0.05) between treatments, n=3.HLSP refers to high light with sufficient P, HLDP refers to high light with deficient P, LLSP refers to low light with sufficient P, and LLDP refers to low light with deficient P.

Supplementary Figure 2. Phenotypic responses of rice plants to exogenous application of root metabolites under low phosphorus conditions.

Supplementary figure. 3 MDA content of two rice cultivars under different light intensities and P supplies.

Vertical bars represent standard error of the mean. Bars labeled with different lowercase letters indicate statistically significant differences (*p*<0.05) between treatments, n=3.HLSP refers to high light with sufficient P, HLDP refers to high light with deficient P, LLSP refers to low light with sufficient P, and LLDP refers to low light with deficient P.


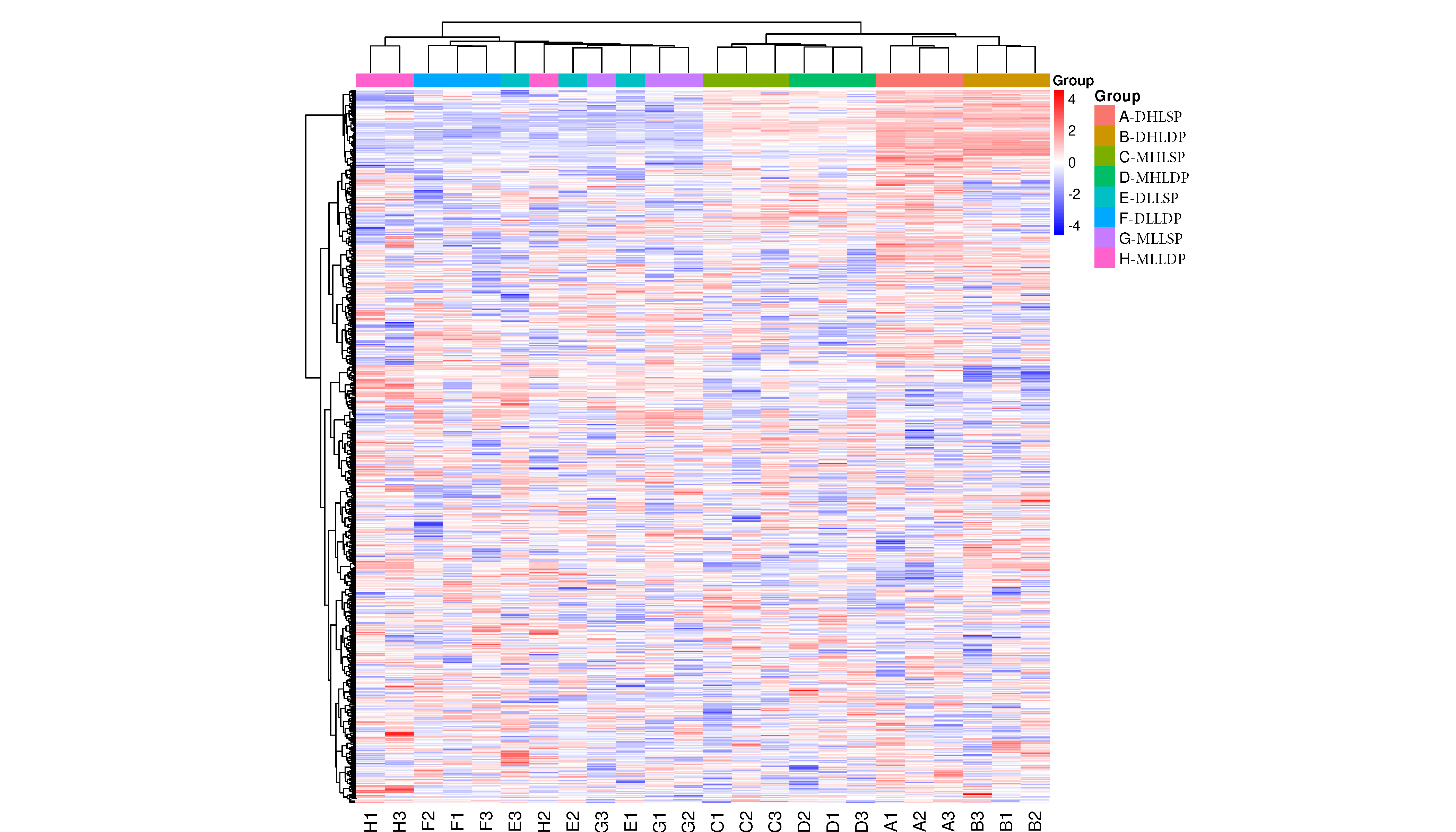


Supplementary figure. 4 The metabolic profiles of several key root exudates across all light intensities and P supplies.

HLSP refers to high light with sufficient P, HLDP refers to high light with deficient P, LLSP refers to low light with sufficient P, and LLDP refers to low light with deficient P. D refers to Dalidao, M refers to Meixiangzhan.


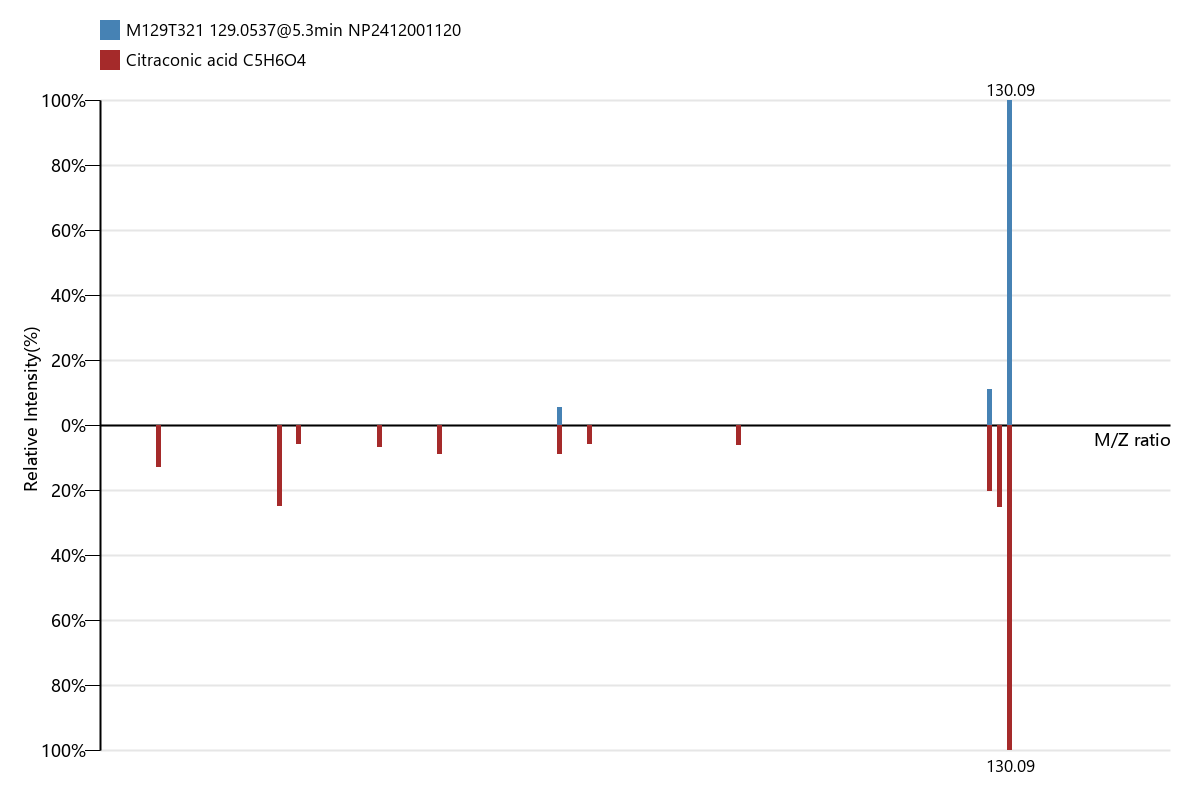


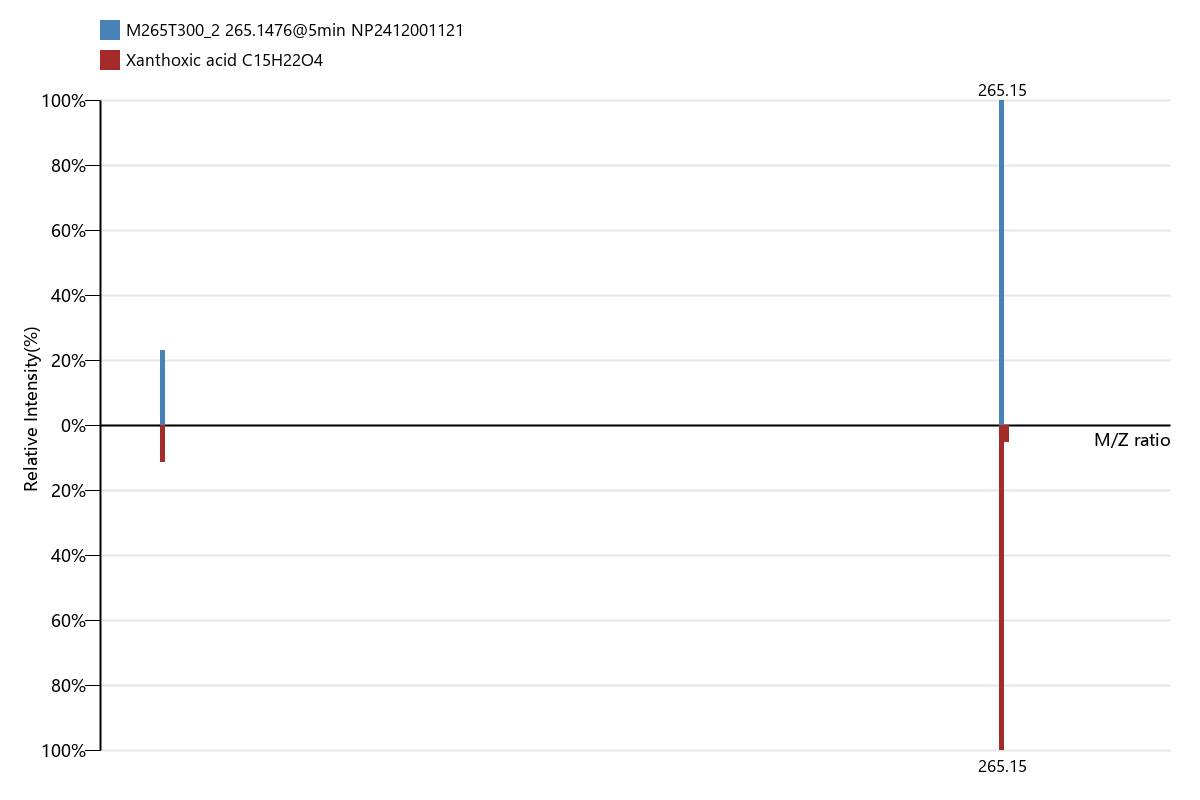


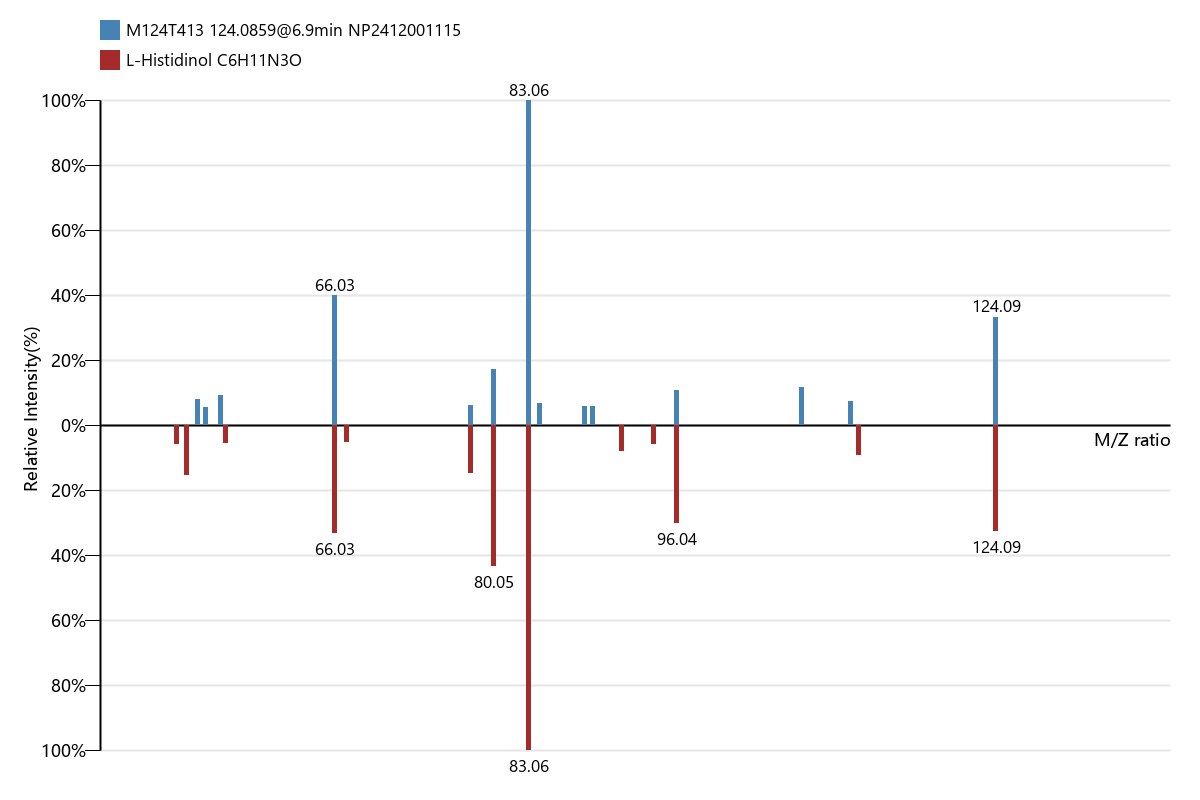


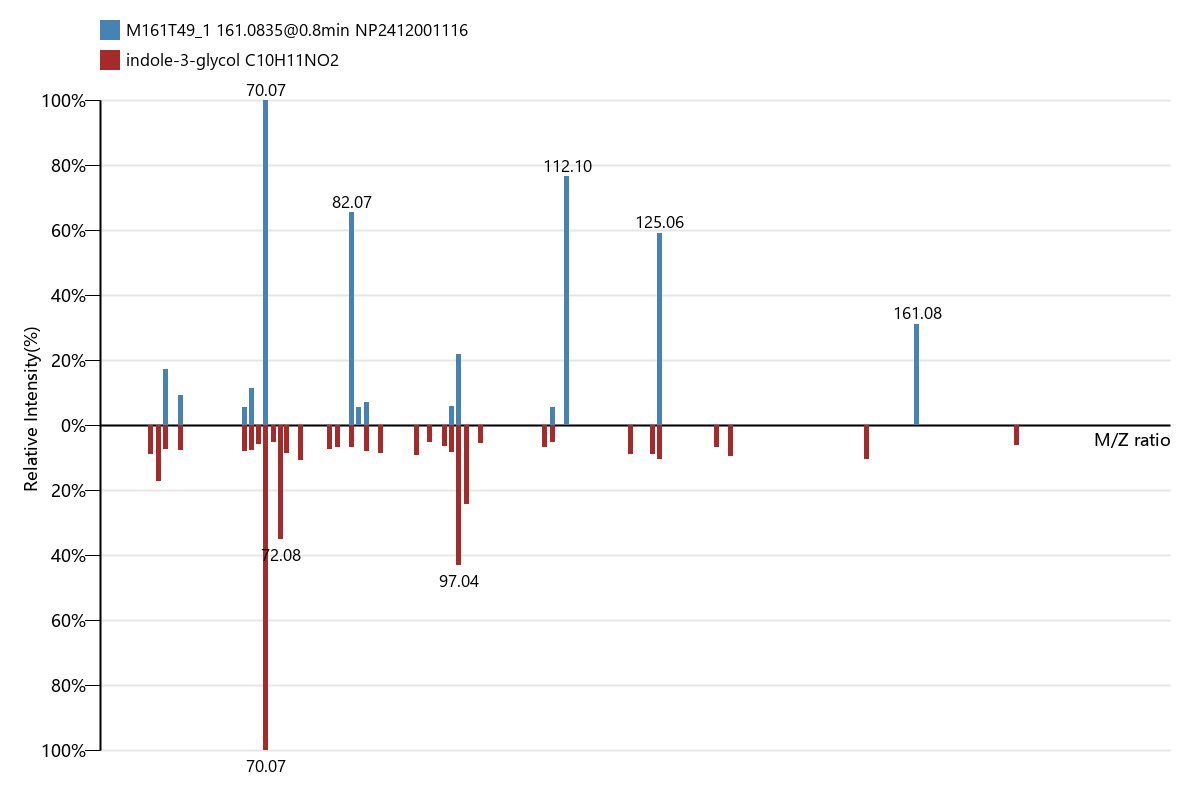


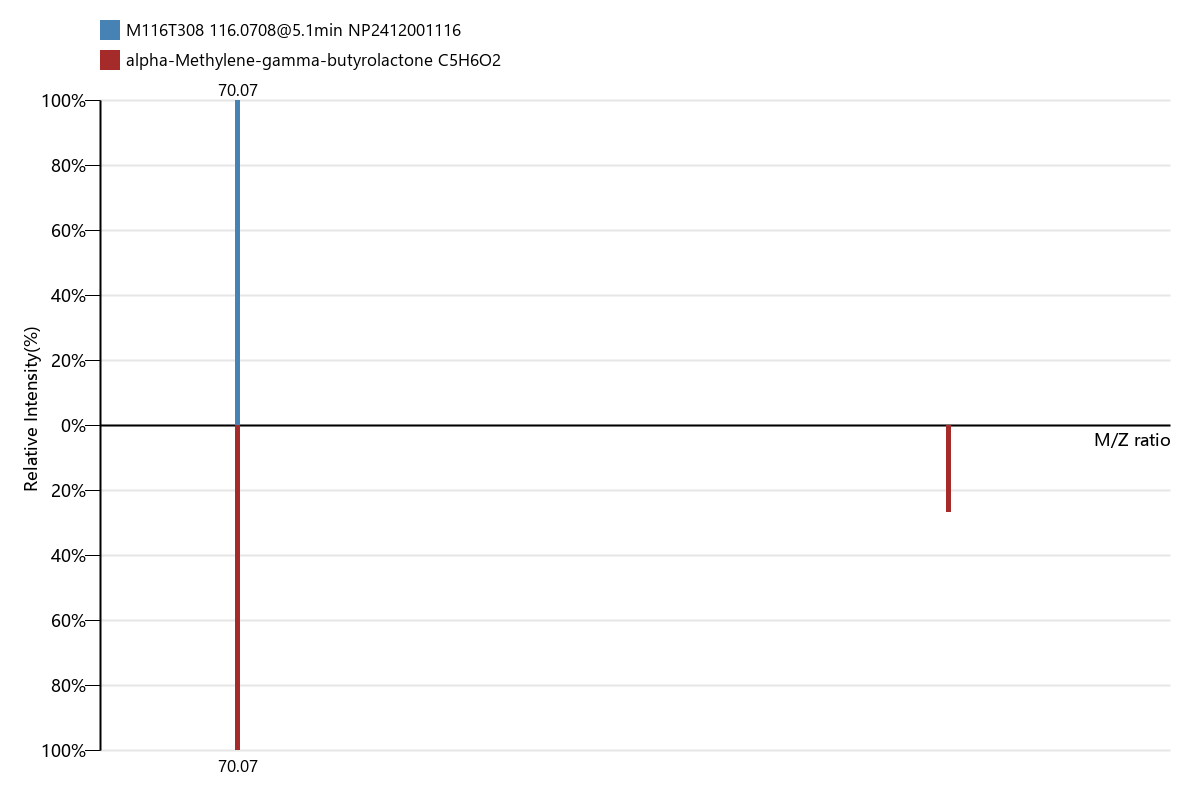


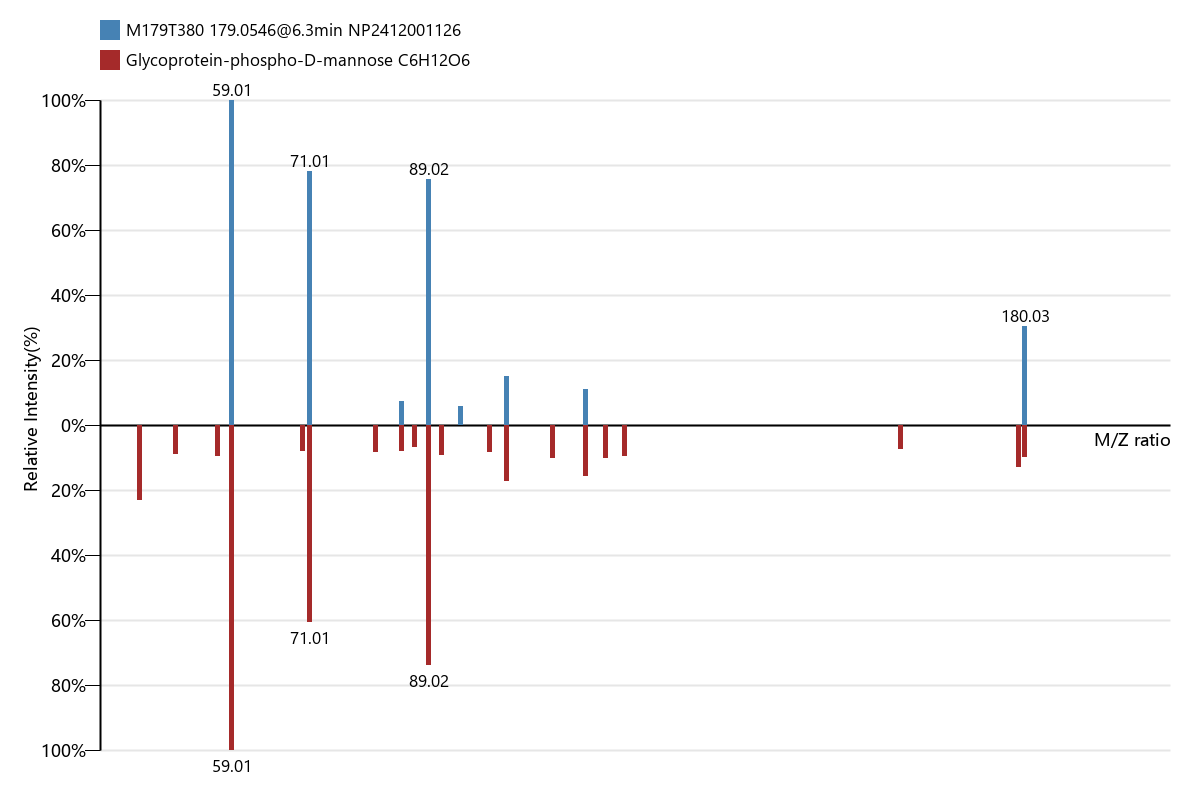

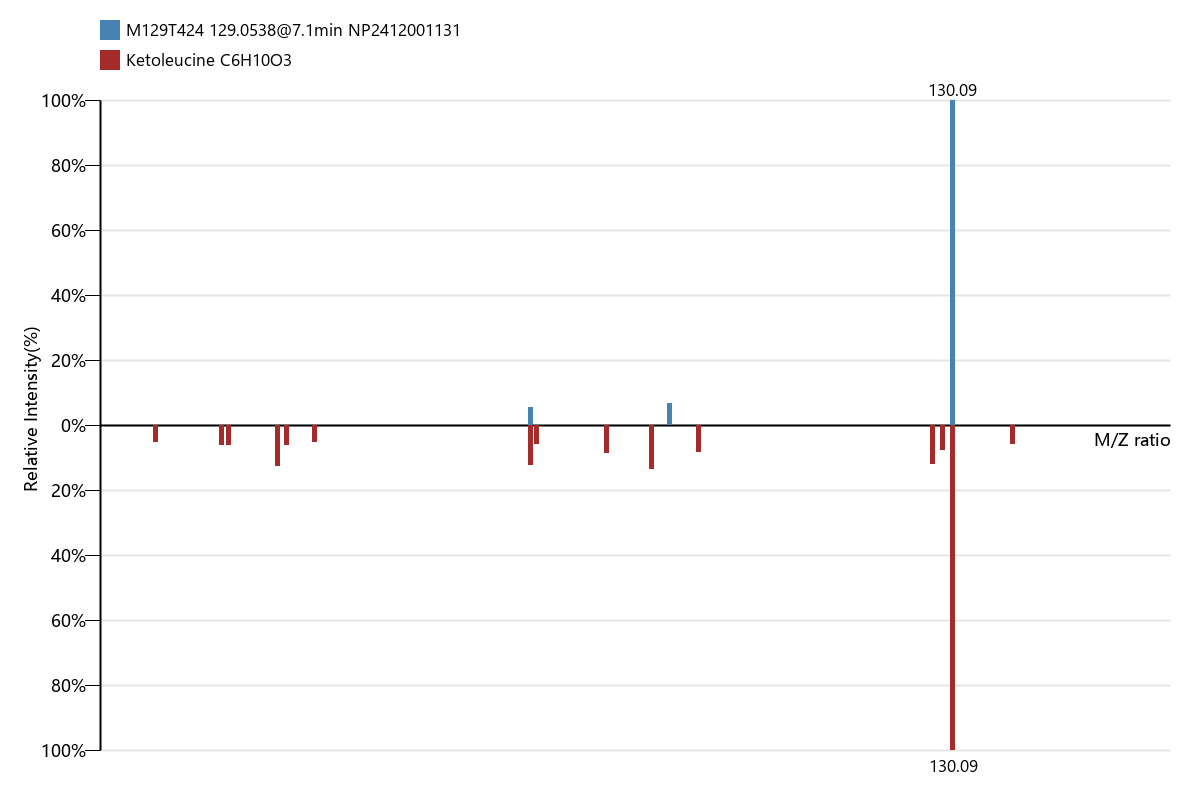


Supplementary figure 5 MS/MS spectra for the seven key metabolites tested functionally (Citraconic acid, Xanthoxic acid, L-Histidinol, indole-3-glycol, alpha-Methylene-gamma-butyrolactone, Glycoprotein-phospho-D-mannose, Ketoleucine)

Supplementary Table 1. F-values of three-factorial analysis of variance on root morphology.

| Treatment | Length  (cm) | Surf Area  (cm^2^) | Avg Diam  (mm) | Root Volume  (cm^3^) | Tips |
| --- | --- | --- | --- | --- | --- |
| Cultivar (C) | 15.273** | 22.608** | 11.24* | 21.867** | 0.103ns |
| Light (L) | 607.384** | 714.234** | 39.387** | 464.677** | 197.439** |
| P supply (P) | 33.392** | 24.221** | 7.569* | 9.169* | 15.246* |
| C×L | 0.003ns | 2.053ns | 0.005ns | 3.495ns | 0.34ns |
| C×P | 0.645ns | 0.533ns | 0.459ns | 0.56ns | 0.316ns |
| L×P | 12.385* | 12.647* | 2.824ns | 5.625* | 2.794ns |
| C×L×P | 0.028ns | 0.008ns | 0.004ns | 0.01ns | 0.289ns |

*Significant at 0.05 probability level. **Significant at 0.01 probability level. ns: not significant.

Supplementary Table 2. Differences in the quantity of root exudates of the phosphorus-sensitive rice cultivar Meixiangzhan (C2) under high light with low phosphorus (HLDP), low light with sufficient phosphorus (LLSP), and low light with low phosphorus (LLDP) treatments.

| Comparison | Up | Down | Total DE |
| --- | --- | --- | --- |
| C2HLSP vs C2HLDP | 35 | 21 | 56 |
| C2LLSP vs C2LLDP | 24 | 38 | 62 |
| C2HLSP vs C2LLSP | 80 | 39 | 119 |
| C2HLDP vs C2LLDP | 48 | 43 | 91 |

**Supplementary Table 3a.** Key differentially expressed root exudates and their metabolic pathways in Dalixiang under high light with sufficient phosphorus (HLSP), high light with deficient phosphorus (HLDP), low light with sufficient phosphorus (LLSP), and low light with low deficient (LLDP) treatments.

| **Comparison** | **Pathway ID** | **Pathway name** | | **Compound name** |
| --- | --- | --- | --- | --- |
| **C1HLSP**  **vs**  **C1HLDP** | osa00940 | Phenylpropanoid biosynthesis | | Sinapic acid; Coniferyl alcohol; Coniferin; Methyleugenol; 5-Hydroxyconiferaldehyde |
|  | osa00750 | Vitamin B6 metabolism | | 5-Pyridoxolactone; (+)-threo-2-Amino-3,4-dihydroxybutanoic acid |
|  | osa00220 | Arginine biosynthesis | | Carbamoyl phosphate; Citrulline |
|  | osa00591 | Linoleic acid metabolism | | Arachidonic acid; 3-HODE + 9-HODE |
|  | osa00620 | Pyruvate metabolism | | DL-Malic acid; 2-Propylmalate |
| **C1LLSP**  **vs**  **C1LLDP** | osa00330 | Arginine and proline metabolism | | (4-Aminobutyl) guanidine; 4-Aminobutyraldehyde; 1-Pyrroline-2-carboxylic acid; 1-Pyrroline-5-carboxylic acid |
|  | osa00290 | Valine, leucine and isoleucine biosynthesis | | L-Threonine; Citraconic acid |
|  | osa00073 | Cutin, suberine and wax biosynthesis | | 9,10-Dihydroxystearic acid |
|  | osa00750 | Vitamin B6 metabolism | | CHEMBL1161469; (+)-threo-2-Amino-3,4-dihydroxybutanoic acid |
|  | osa00770 | Pantothenate and CoA biosynthesis | | Uracil; Panthenol |
| **C1HLSP**  **vs**  **C1LLSP** | osa00250 | Alanine, aspartate and glutamate metabolism | Oxoglutaric acid; Succinic acid; L-Aspartic acid; Carbamoyl phosphate | |
|  | osa00020 | Citrate cycle (TCA cycle) | Oxoglutaric acid; Succinic acid; Aconitate [cis or trans] | |
|  | osa00220 | Arginine biosynthesis | Oxoglutaric acid; L-Aspartic acid; Carbamoyl phosphate | |
|  | osa00310 | Lysine degradation | Oxoglutaric acid; Succinic acid; L-Lysine; Glutaric acid; Nα-Acetyl-L-lysine | |
|  | osa00470 | D-Amino acid metabolism | Oxoglutaric acid; L-Lysine; L-Aspartic acid; L-Proline; Phenylpyruvate | |
| **C1HLDP**  **vs**  **C1LLDP** | osa00340 | Histidine metabolism | L-Glutamic acid; Histamine; Urocanic acid; L-Histidinol; Histidinal | |
|  | osa00220 | Arginine biosynthesis | L-Glutamic acid; L-Arginine; Carbamoyl phosphate | |
|  | osa00750 | Vitamin B6 metabolism | CHEMBL1161469; 5-Pyridoxolactone; (+)-threo-2-Amino-3,4-dihydroxybutanoic acid | |
|  | osa00410 | beta-Alanine metabolism | Uracil; 4-Aminobutyraldehyde; Propynoic acid | |
|  | osa00970 | Aminoacyl-tRNA biosynthesis | L-Glutamic acid; L-Lysine; L-Arginine; L-Tryptophan | |

**Supplementary Table 3b.** Key differentially expressed root exudates and their metabolic pathways between Dalixiang and Meixiangzhan cultivars under high light with sufficient phosphorus (HLSP), high light with deficient phosphorus (HLDP), low light with sufficient phosphorus (LLSP), and low light with deficient phosphorus (LLDP) treatments.

| **Comparison** | **Pathway ID** | **Pathway name** | | **Compound name** |
| --- | --- | --- | --- | --- |
| **C1HLSP**  **vs**  **C2HLSP** | osa00660 | C5-Branched dibasic acid metabolism | | L-Glutamic acid; Aconitate [cis or trans]; (R)-Acetoin; Citraconic acid |
|  | osa00410 | beta-Alanine metabolism | | 4-Aminobutyraldehyde; Propynoic acid; Quinolinic acid |
|  | osa00910 | Nitrogen metabolism | | L-Glutamic acid; Carbamoyl phosphate |
|  | osa00960 | Tropane, piperidine and pyridine alkaloid biosynthesis | | L-Lysine; L-Isoleucine; Trigonelline (N'-methylnicotinate); L-3-Phenyllactic acid |
|  | osa00290 | Valine, leucine and isoleucine biosynthesis | | L-Isoleucine; Citraconic acid |
| **C1HLDP**  **vs**  **C2HLDP** | osa00220 | Arginine biosynthesis | | L-Glutamic acid; Oxoglutaric acid; L-Arginine; L-Glutamine; Carbamoyl phosphate |
|  | osa00340 | Histidine metabolism | | L-Glutamic acid; Oxoglutaric acid; Histamine; Urocanic acid; L-Histidinol; Histidinal |
|  | osa00250 | Alanine, aspartate and glutamate metabolism | | L-Glutamic acid; Oxoglutaric acid; L-Glutamine; Carbamoyl phosphate |
|  | osa00630 | Glyoxylate and dicarboxylate metabolism | | L-Glutamic acid; Oxoglutaric acid; L-Glutamine; DL-Malic acid; D-Glycerate 3-phosphate; Aconitate [cis or trans] |
|  | osa00910 | Nitrogen metabolism | | L-Glutamic acid; L-Glutamine; Carbamoyl phosphate |
| **C1LLSP**  **vs**  **C2LLSP** | osa00290 | Valine, leucine and isoleucine biosynthesis | L-Threonine; Citraconic acid | |
|  | osa00330 | Arginine and proline metabolism | (4-Aminobutyl) guanidine; Aspartate semialdehyde; 1-Pyrroline-5-carboxylic acid, | |
|  | osa00261 | Monobactam biosynthesis | L-Threonine; Aspartate semialdehyde | |
|  | osa00592 | alpha-Linolenic acid metabolism | FAL 6_1; 10-OPDA | |
|  | osa00260 | Glycine, serine and threonine metabolism | L-Threonine; Aspartate semialdehyde | |
| **C1LLDP**  **vs**  **C2LLDP** | osa00750 | Vitamin B6 metabolism | Pyridoxine; (+)-threo-2-Amino-3,4-dihydroxybutanoic acid | |
|  | osa00770 | Pantothenate and CoA biosynthesis | Panthenol; (S)-2-Acetolactate | |
|  | osa00330 | Arginine and proline metabolism | (4-Aminobutyl) guanidine; 4-Aminobutyraldehyde; N-Acetylputrescine | |
|  | osa00340 | Histidine metabolism | Histamine; Urocanic acid | |
|  | osa00360 | Phenylalanine metabolism | 4-hydroxyphenylacetate; Hydrocinnamic acid | |
